# The expression and significance of mTORC1 in diabetic retinopathy

**DOI:** 10.1186/s12886-020-01553-3

**Published:** 2020-07-20

**Authors:** Yanli Liu, Yarong Zheng, Yekai Zhou, Yi Liu, Mengxuan Xie, Wenjing Meng, Meixia An

**Affiliations:** 1 The Third Affiliated Hospital of Southern Medical University, Guangzhou, 510515 China; 2 Guangdong Provincial Key Laboratory of Bone and Joint Degeneration Diesases, Guangzhou, China

**Keywords:** Diabetic retinopathy, mTORC1, P-S6, VEGF, PEDF, Cell proliferation and migration

## Abstract

**Background:**

To investigate the expression and significance of mechanistic target of rapamycin complex 1(mTORC1) in diabetic retinopathy (DR), and to find new targets and new methods for the treatment of DR.

**Methods:**

A DR rat model was prepared by general feeding combined with intraperitoneal injection of 10% streptozotocin (60 mg/kg). The rats were randomly divided into a control group (NDM group) and a diabetes group (DM group). Three months later, the degrees of retinopathy was determined using hematoxylin and eosin staining, and the levels of p-S6, VEGF, and PEDF proteins were detected by immunohistochemistry and western blotting. Human retinal capillary endothelial cells (HRCECs) were cultured in high glucose (HG) conditions, then treated with rapamycin or transfected with siTSC1.The protein levels of p-S6 were assessed by western blotting. The 5-ethynyl-2′-deoxyuridine assay was used to detect cell proliferation, and the Transwell assay was used to detect cell migration.

**Results:**

A DM rat model was successfully developed. The expressions of p-S6 and VEGF proteins were significantly increased in the DM group (*p* < 0.05), and the expression of PEDF protein was significantly decreased compared with the NDM group (*p* < 0.05). In vitro, the p-S6 protein, as well as cell proliferation and migration, in HG induced HRCECs were increased (*p* < 0.05) compared with the control (normal glucose) group (p < 0.05). After transfection with siTSC1 to activate mTORC1, the expression of p-S6, as well as cell proliferation and migration, were increased. In contrast, rapamycin decreased p-S6 expression, as well as proliferation and migration, in HG induced HRCECs compared to the control group (*p* < 0.05).

**Conclusion:**

mTORC1 plays an important role in DR. After activation, mTORC1 induced expression of the p-S6 protein, regulated the expressions of VEGF and PEDF proteins, and changed the proliferation and migration of endothelial cells. The mTORC1 can therefore be used as a new target,as well as in the treatment of DR.

## Background

Diabetic retinopathy (DR), which is a microvascular complication of diabetes mellitus (DM), is the most common retinal vascular disease, and is one of the major blinding eye diseases for patients over 50 years of age [1]. However, the present treatment options for DR are limited [2]. Studies have confirmed that the destruction of pancreatic beta-cells in a high glucose (HG) environment leads to DM [3]. The retina is in a HG environment for a long time, which leads to the destruction of the blood-retinal barrier, and induction of excessive production of pro-inflammatory cytokines, such as TNF-α,and IL-1β [4], which stimulate excessive production of reactive oxygen species in the mitochondrial electron transfer chain, to cause oxidative stress and to limit the production of energy [5], resulting in the inhibition of cell autophagy [6]. This series of processes eventually leads to retinopathy [7], promoting the release of a large number of cytokines [8], which leads to the occurrence of DR.

The mammalian target of rapamycin (mTOR) is a highly conserved serine/threonine protein kinase in structure and function, involving mTORC1, and is a central signaling molecule that integrates various pathways inside and outside cells, regulates cell growth and metabolism, and provides an important molecular link between nutritional signals and metabolic processes necessary for cell growth. It mainly promotes cell growth, proliferation and differentiation by activating key anabolic processes. Improper regulation of mTORC1 is the basis of many human diseases, including cancer, diabetes, autoimmune diseases, and nervous system diseases [9]. Several recent studies have demonstrated that mTOR may play a vital role in DR pathophysiology. The research by Calton and Vollrath proved that inhibition of mTOR reduced migration of retinal pigment epithelial cells [10]. Fort and colleagues found that mTORC1 caused an independent reduction of retinal protein synthesis in type 1 diabetes. However, the effect of mTORC1 activation on DR development has not been reported [11]. The roles of mTORC1 in aberrant endothelial cell proliferation and migration, and the crucial events in DR progression, as well as its underlying mechanisms, are not known.

In the present study, the rat DR model and the human retinal capillary endothelial cells (HRCECs) HG model were constructed. To determine the role of the mTORC1 signaling pathway in the pathogenesis of DR, as well as to find new targets and methods of treatment for this disorder, we first measured the expression level of mTORC1 downstream phospo-S6 ribosomal protein (p-S6). We thenanalyzed its relationship with the expression level of vascular endothelial growth factor (VEGF) and recombinant human pigment epithelium-derived factor (PEDF), and characterized retinal proliferation and migration.

## Methods

### Animals

Healthy male SPF SD rats, weighing 200 ± 20 g(*n* = 12),were obtained from the Animal Laboratory Center, Southern Medical University [license No. SCXK (Guangdong, China) 2016–0041]. All experimental animals were fed and followed-up in the animal room of the Medical Research Center of the Third Affiliated Hospital of Southern Medical University. The experimental protocols were approved by the Animal Ethics Committee of Southern Medical University.

### Diabetes induction

The rats were randomly divided into a non-diabetes mellitus (NDM) group and diabetes mellitus (DM) group. After adaptive feeding for 3 days, the DM group(*n* = 6) was injected intraperitoneally with 10% streptozotocin (STZ) at a dose of 60 mg/kg body weight, STZ was dissolved in a citric acid-sodium citrate buffer solution of 0.1 mmol/L and pH of 4.4, which was protected from light and placed on ice [12, 13]. The NDM group(*n* = 6) received the same dose of citric acid-sodium citrate buffer. In the DM model rats, animals with blood sugar ≥16.7 mmol/L were used for tail venous blood tests. All animals were euthanized at 3 months by intraperitoneal injection of pentobarbital sodium, and their blood samples and retinas were harvested for protein preparation, and the eye balls were removed for paraffin sections.

### Major reagents

STZ, citric acid, and sodium citrate (Sigma-Aldrich, St. Louis, MO, USA), anti-VEGF mouse monoclonal antibody (Abcam, Cambridge, MA, USA),1:3000 [14, 15], anti-PEDF rabbit polyclonal antibody (ABclonal, Wuhan, China),1:3000,anti-PS6 rabbit monoclonal antibody (Ser235/236; Cell Signaling Technology, Danvers, MA, USA)1:1500 [16], anti-S6 rabbit polyclonal antibody (Cell Signaling Technology),1:5000 [17], anti β-tubulin mouse monoclonal antibody (Beijing Kangwei Century Biotechnology, Beijing, China;1:5000), anti-mouse secondary antibody and anti-rabbit secondary antibody (Beijing Ruikang Biotechnology, Beijing, China;1:3000), Endothelial Cell Medium (ECM; Sciencell, Carlsbad, CA, USA), and rapamycin (APExBIO; Boston, MA, USA) were used in this study.

### Trypsin digest preparation of retinal vasculature

Retinal vasculature was prepared according to the protocol written by Jonathan C. Chou [18] Briefly, the eyeballs were enucleated and fixed in 4% paraformaldehyde for 24 h. The retinas were dissected from the eyeballs, washed in water overnight, and digested in 3% trypsin (Solarbio, Beijing, China) for 2 h at 37 °C. The tissues were repeatedly washed in water to remove debris, transfered onto clean slides and unfolded under a dissection microscope, stained with hematoxylin & eosin (Solarbio, Beijing, China) after natural drying, then dehydrated and mounted. The prepared retinal vessels were photographed by microscope and 5 pictures were selected from each group. The number of endothelial cells and pericytes from images were counted and the ratio(E/P) was calculated to assess the degree of retinopathy.

### Cell culture

HRCECs were from Guangzhou Jennio Biotechnology (Guangzhou, China) and were cultured in media supplemented with 1% endothelial cell growth supplement, 1% penicillin/streptomycin solution, and 5% fetal bovine serum (FBS) at 37 °C, and were incubated in a humidified incubator with 5% CO_2_. When HRCECs reached 80–90% confluence, they were digested with 0.25% trypsin without EDTA and passed at a ratio of 1:2. The concentration of glucose in the ECM was 1 g/L (5.5 mmol/L), which was similar to the normal blood glucose concentration of the human body. HRCECs were cultured in the same normal glucose concentration as the control group (NG group). D-(+)- glucose was added to the ECM, and the final concentration of glucose was adjusted to 4.5 g/L (25 mmol/L) to simulate the diabetic microenvironment of the human body, which was used to develop a HRCEC HG model (HG group) [19]. The final concentration of rapamycin (inhibitor of the mTORC1 pathway) in HG-ECM used to treat HRCECs was 50 nM (HG + rapamycin). Cells treated under different conditions were placed in a constant temperature incubator for culturing, and the medium was changed every day for subsequent protein extractions.

### Transfection

The siTSC1 (sense, 5′-CCAAAUCUCAGCCCGCUUUTT-3′ and antisense, 5′-AAAGCGGGCUGAGAUUUGGTT-3′) were purchased from Sangon Biotech (Shanghai, China). They were separately transfected into HRCECs using Lipofectamine 3000 reagent (Life Technologies, Carlsbad, CA, USA) according to the manufacturer’s instructions.

### Transwell assay

After HRCECs were treated with different interventions for 48 h, 1 × 10^5^ cells/mL were diluted with serum-free ECM and transferred into the upper chamber of a Transwell insert (Corning, Corning, NY, USA). ECM containing 5% FBS was added to the lower chamber. After 24 h of incubation in a CO_2_ incubator at 37 °C, the non-migrating cells were gently removed from the upper chamber. Cells that had migrated through the membrane were fixed with 4% paraformaldehyde for 20 min and stained with a 0.5% Crystal Violet solution for 10 min. The migrated cells were imaged using an inverted optical microscope, and five fields of view were randomly selected to count cell numbers.

### 5-ethynyl-2′-deoxyuridine (EdU) assay

HRCECs were inoculated into 15 mm glass-bottomed dishes. After becoming adherent and reaching 60–70% confluency after 6 h, they were separately treated with different interventions. After 48 h of treatment, the cell proliferative capacity was assayed with a kFluor488-EdU assay kit (KeyGen, Nanjing, China) according to the manufacturer’s instructions. Samples were incubated with 50 μM EdU working solution for 2 h. The cells were then imaged using a fluorescence microscope. Five fields of view were randomly selected to calculate the positive rate.

### Statistical method

All experiments were performed in triplicate and observed by independent observations. SPSS statistical software for Windows, version 19.0 (SPSS, Chicago, IL, USA) was used for statistical analysis. The experimental data are expressed as the average number ± standard deviation (x ± s). Each test sample was provided with two secondary wells. The *t*-test was used for comparison between two groups. The difference was statistically significant with a value of *p* < 0.05.

## Results

### Establishment of a diabetic rat model

**Fig. 1:**
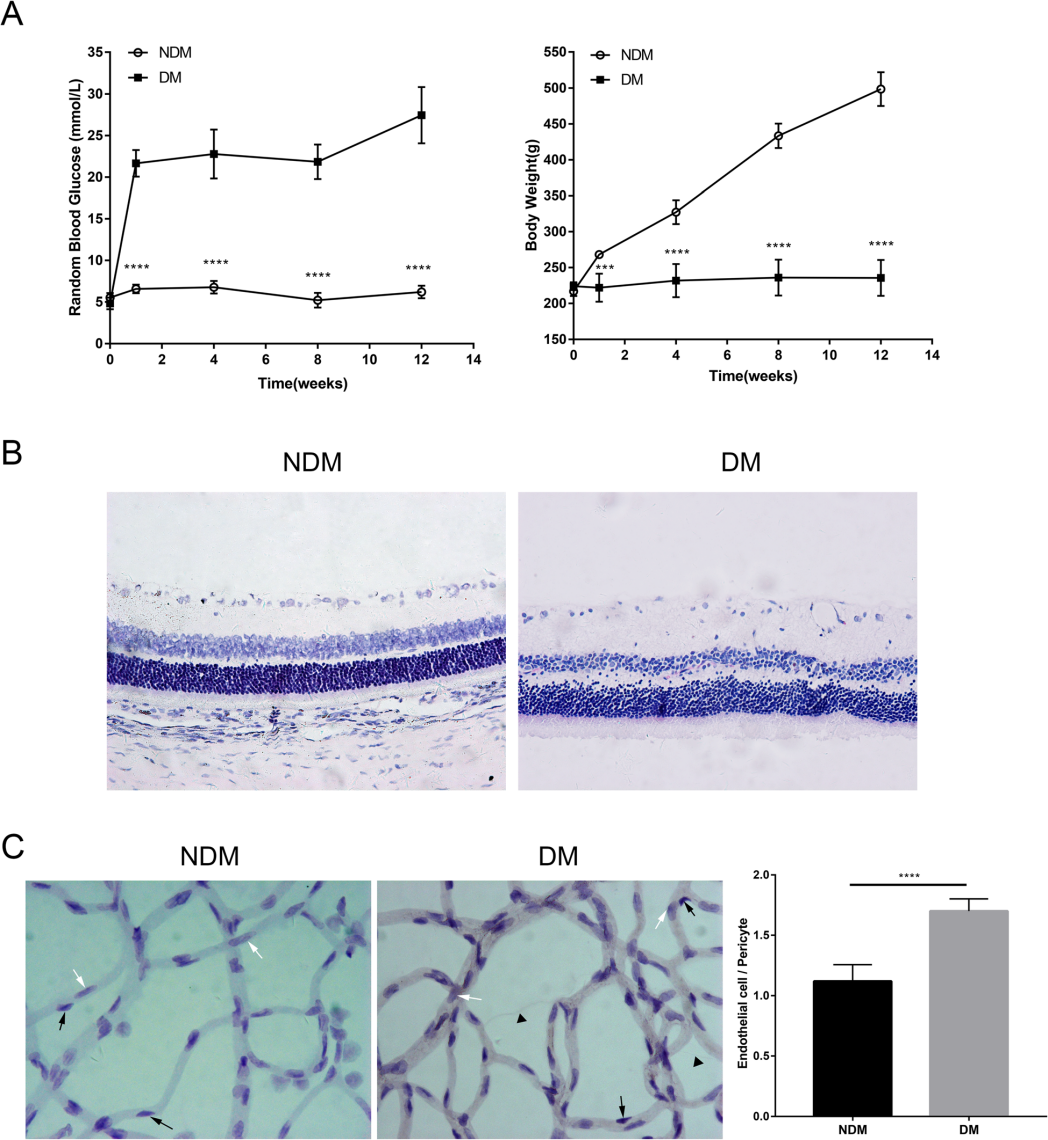
The successful establishment of a retinopathy model in streptozotocin-induced diabetic rats. a Random blood glucose levels and body weights of rats. **b** H*ematoxylin* and eosin staining of retinal paraffin sections (200×). NDM, non-diabetes mellitus; DM, diabetes mellitus. **c** The prepared retinal vasculature by trypsin digest (400x). Endothelial cell (white arrow), pericyte (black arrow), and acellular capillaries (triangle)

In the NDM group, the blood glucose level of rats was always≤16.7 mmol/L. Before eating and drinking the same amount, the rats gradually gained weight and had clean and shiny fur. After 1 week, the blood glucose levels of the DM group rats were all ≥16.7 mmol/L. The blood glucose level of the DM group rats was significantly increased, the intake of food was increased, and the amount of drinking water was increased, with approximately twice that of the healthy rats. However, weight gain was not obvious in DM rats within 3 months, and several even lost weight, with sallow fur and bent backs (Fig. 1a).

In NDM rat retinas, the structures of each layer were clear and distinct after hematoxylin and eosin staining. In the DM group, the structure of the retinal layers was disordered, the inner limiting membrane was swollen, dilated blood vessels could be seen in the ganglion cell layer, and more blood vessels could be seen in the outer plexiform layer (Fig. 1b).

As showed in the retinal vasculature, the nucleuses of endothelial cells were big, oval or irregular which paralleled with the vasculature in long axis (Fig. 1c, white arrow); the nucleuses of pericytes were small and round, being triangle, located at one side of the capillary (Fig. 1c, black arrow). In DM rat, the ratio of the number of endothelial cells to the number of pericytes(E/P) was significantly increased compared with NDM rat(p<0.0001, Fig. 1c).NDM rat showed normal vascular architecture, while diabetic rat retinas exhibited increased acellular capillaries (Fig. 1c, triangle).

### mTORC1 was highly activated in retinas of diabetic rats when compared with nondiabetic rats

**Fig. 2:**
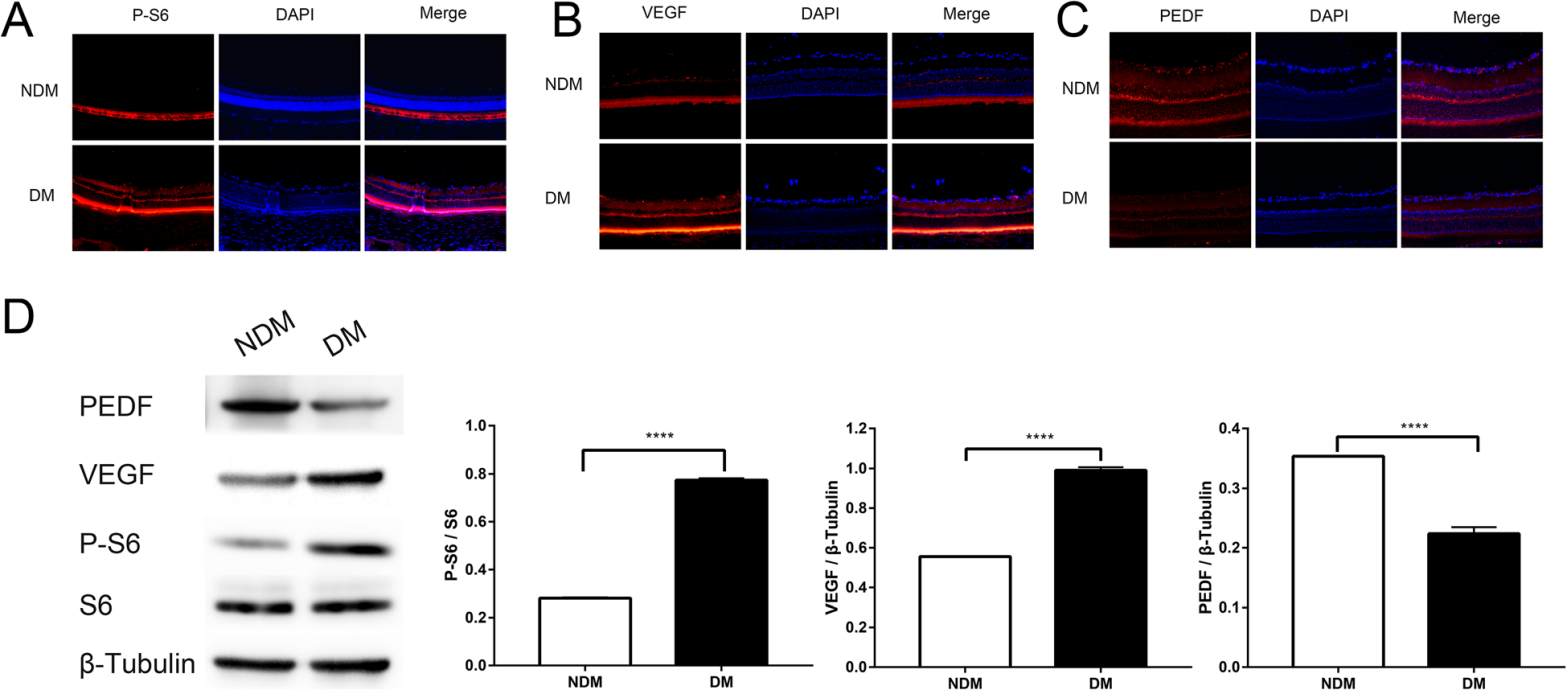
mTORC1 is highly activated in retinas of diabetic rats. **a** Retinal immunofluorescence staining of p-S6 protein. **b** The retinal immunofluorescence staining of VEGF protein. **c** Retinal immunofluorescence staining of PEDF protein. **d** The protein contents of p-S6, VEGF, and PEDF in rat retinas. Values are mean ± 95%CI. ^****^p < 0.0001

Rats were euthanized 12 weeks after successful modeling, and retinal tissues were obtained. We next determined whether mTOR was activated in diabetic rats compared with non-diabetic rats. Immunofluorescence analysis revealed significantly upregulated phosphorylation of S6 (S235/236; a downstream effector of mTORC1 and S6K1) expression in retinas of diabetic rats, when compared with non-diabetic rats (Fig. 2a). We also found that VEGF was highly expressed in the retinas of diabetic rats, while PEDF was significantly decreased in the retinas of diabetic rats, when compared with non-diabetic rats (Fig. 2b, c). These results were confirmed by western blotting (Fig. 2d). Together, these results showed that mTOR signaling, mTORC1 in particular, was highly activated in retinas of diabetic rats.

### Rapamycin inhibited the proliferation and migration of HG induced HRCECs

**Fig. 3:**
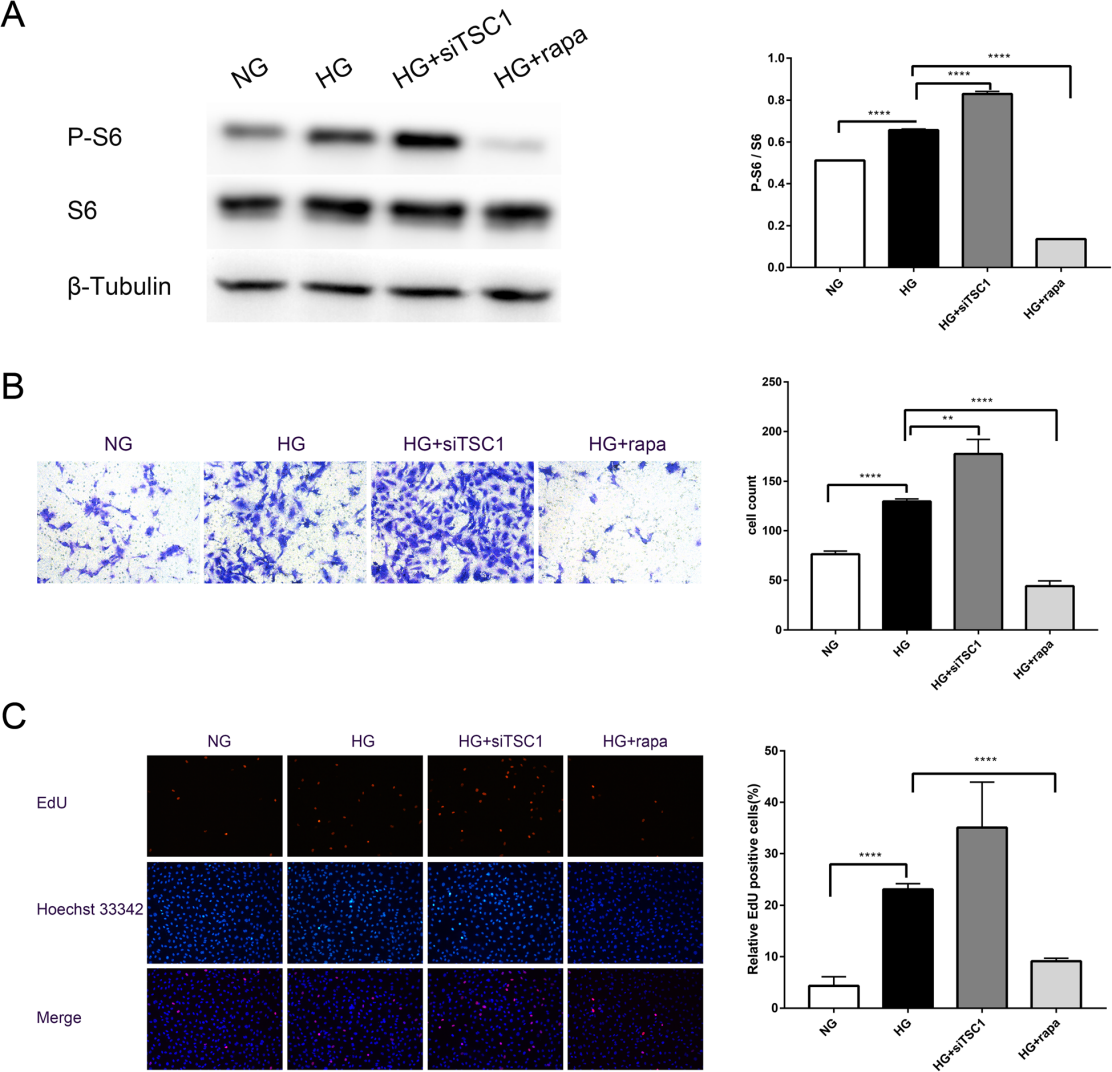
Rapamycin inhibits the proliferation and migration of HG-induced human retinal capillary endothelial cells (HRCECs). **a** The p-S6 expression in HRCECs transfected with siTSC1 or rapamycin. **b** The migration ability of HRCECs assayed using the Transwell assay. **c** The proliferation ability of HRCECs assayed by 5-ethynyl-2′-deoxyuridine. Values are mean ± 95% CI. ^**^p < 0.01; ^****^p < 0.0001

Western blotting showed that the p-S6 contents were higher in HG-HRCECs than the NG group (Fig. 3a). In HG-HRCECs transfected with siTSC1, the protein contents of p-S6 were higher than the HG control, and after treatment with rapamycin, the p-S6 contents were decreased in HG-HRCECs. In the Transwell assay, HG increased the migration ability of HRCECs, which could be transfected with siTSC1. After treatment with rapamycin, the migration ability decreased (Fig. 3b). In the EdU assay, HG treatment induced a significant increase in the proliferation of HRCECs compared with the NG group, while treatment with rapamycin inhibited HG-induced proliferation (Fig. 3c).

## Discussion

mTOR was first discovered by Heirman and others when analyzing the difference of resistance of beer yeast mutants to rapamycin [20]. The mTOR is a serine/threonine protein kinase,which is highly conserved in structure and function, belonging to a phosphatidylinositol 3-kinase (PI3K)-related family member [21]. mTOR mainly exists in the form of two complexes in vivo: mTORC1, which regulates cell proliferation and metabolic reactions and mTORC2. Abnormalities in any link can change mTORC1 activity [22], leading to the development of diseases, such as diabetes, cancer, and aging [23]. At present, a large number of studies have confirmed that the PI3K/Akt/mTOR signaling pathway is related to some complications of diabetes, such as diabetic nephropathy [24], neuropathy [25], and myocardial ischemia [26]. We therefore suspected that the occurrence and development of DR was related to mTORC1 activation. The mTORC1 is mainly composed of mTORC (core protein), RAPTOR (scaffold protein), DEPTOR (endogenous kinase inhibitor), PRAS40 (endogenous kinase inhibitor), and mLST8 [27]. There is a dephosphorylated S6K downstream of the mTORC1 pathway, which is located on the eIF3 scaffold complex. Active mTORC1 is recruited onto the eIF3 scaffold and then phosphorylates S6K to activate it [28]. The mTORC1 phosphorylates at least two amino acid residues of S6K1, of which the most critical modification is located on the threonine residue (T389) [29].S6K1 activity can be determined by activating S6 ribosomal protein (phospo-S6 ribosomal protein, p-S6) and eIF4B, and the level of P-S6 in the body can be used as an indicator of the activation degree of the mTORC1 pathway [30].

In the present study, we used a STZ -induced rat model, which is frequently used for studies on diabetes and its complications. We found that the retinal structures of DM rats were more disordered than NDM rats, and that there were more blood vessels in the retinas of DM rats, which was consistent with the results of other studies. We also found that p-S6 and VEGF proteins were significantly increased in the DM group, and the expression of PEDF protein was significantly decreased compared with the control group. These results suggested that activation of the mTORC1 pathway may exist in DR. DR involves microangiopathy, and vascular endothelial cells are the primary cellular targets in DR. Thus, HRCECs were cultured in ECM with 25 mmol/L glucose to simulate the diabetic microenvironment. In vitro, the p-S6 protein in HG-induced HRCECs was increased compared with the control (normal glucose)group. After transfection with siTSC1 to activate mTORC1, the expression level of p-S6 was increased, and the processes of proliferation and migration were increased, whereas rapamycin decreased the p-S6 expression in HG-induced HRCECs, and the processes of proliferation and migration were also decreased.

In summary, mTORC1 played an important role in DR. It was activated in DR to produce the p-S6 protein, to regulate the expressions of VEGF and PEDF proteins, and to change the proliferation and migration of endothelial cells, which are the main characters of DR development. However, it is also clear that in vitro cell line expression systems do not fully replicate the vivo cellular environment. Therefore, future studies in DR rats using rapamycin will be performed to confirm the effect of mTORC1 during DR development and to explore the appropriate therapeutic dose of rapamycin.

## Conclusion

mTORC1 may be a new target for the treatment of diabetic retinopathy, and its specific inhibitors (such as rapamycin) may also provide a novel means of treatment.

## Data Availability

The datasets during and analysed during the current study available from the corresponding author on reasonable request.

## Abbreviations

mTOR: Mammalian target of rapamycin
mTORC1: Mechanistic target of rapamycin complex 1
DM: Diabetes mellitus
DR: Diabetic retinopathy
HG: High glucose
HRCECs: Human retinal capillary endothelial cells
p-S6: Phospo-S6 ribosomal protein
VEGF: Vascular endothelial growth factor
PEDF: Pigment epithelium-derived factor
STZ: Streptozotocin
FBS: Fetal bovine serum
EdU: The 5-ethynyl-2′-deoxyuridine
